# Divergent Evolution of CHD3 Proteins Resulted in MOM1 Refining Epigenetic Control in Vascular Plants

**DOI:** 10.1371/journal.pgen.1000165

**Published:** 2008-08-22

**Authors:** Marian Čaikovski, Chotika Yokthongwattana, Yoshiki Habu, Taisuke Nishimura, Olivier Mathieu, Jerzy Paszkowski

**Affiliations:** 1 Laboratory of Plant Genetics, University of Geneva, Geneva, Switzerland; 2 National Institute of Agrobiological Sciences, Tsukuba, Ibaraki, Japan; 3 Division of Biological Sciences, Graduate School of Science, Hokkaido University, Sapporo, Japan; 4 Centre National de la Recherche Scientifique (CNRS), UMR6247 - INSERM U931 - GReD, Clermont Université, Aubière Cedex, France; National Institute of Genetics, Japan

## Abstract

*Arabidopsis* MOM1 is required for the heritable maintenance of transcriptional gene silencing (TGS). Unlike many other silencing factors, depletion of MOM1 evokes transcription at selected loci without major changes in DNA methylation or histone modification. These loci retain unusual, bivalent chromatin properties, intermediate to both euchromatin and heterochromatin. The structure of MOM1 previously suggested an integral nuclear membrane protein with chromatin-remodeling and actin-binding activities. Unexpected results presented here challenge these presumed MOM1 activities and demonstrate that less than 13% of MOM1 sequence is necessary and sufficient for TGS maintenance. This active sequence encompasses a novel Conserved MOM1 Motif 2 (CMM2). The high conservation suggests that CMM2 has been the subject of strong evolutionary pressure. The replacement of *Arabidopsis* CMM2 by a poplar motif reveals its functional conservation. Interspecies comparison suggests that MOM1 proteins emerged at the origin of vascular plants through neo-functionalization of the ubiquitous eukaryotic CHD3 chromatin remodeling factors. Interestingly, despite the divergent evolution of CHD3 and MOM1, we observed functional cooperation in epigenetic control involving unrelated protein motifs and thus probably diverse mechanisms.

## Author Summary

Epigenetic regulation of transcription usually involves changes in histone modifications, as well as DNA methylation changes in plants and mammals. Previously, we found an exceptional epigenetic regulator in *Arabidopsis*, MOM1, acting independently of these epigenetic marks. Interestingly, MOM1 controls loci associated with bivalent chromatin marks, intermediate to active euchromatin and silent heterochromatin. Such bivalent marks are often associated with newly inserted and/or potentially active transposons, silent transgenes, and certain chromosomal loci. Notably, bivalent chromatin seems to be characteristic for embryonic stem cells, where such loci change their activity and determination of epigenetic marks during cell differentiation. Here, we provide evidence that in vascular plants, the MOM1-like proteins evolved from the ubiquitous eukaryotic chromatin remodeling factor CHD3. The domains necessary for CHD3 function degenerated in MOM1, became dispensable for its gene silencing activity, and were replaced by a novel, unrelated domain providing silencing function. Therefore, MOM1-like proteins use a different silencing mechanism compared to the ancestral CHD3s. In spite of this divergent evolution, CHD3 and MOM1 seem to retain a functional cooperation in control of transcriptionally silent loci. Our results provide an unprecedented example of an evolutionary path for epigenetic components resulting in increased complexity of an epigenetic regulatory network characteristic for multicellular eukaryotes.

## Introduction

TGS heritably suppresses transcription of repetitive elements, transgenes and chromosomal genes and is generally associated with repressive histone marks and hypermethylation of DNA. Mutations in *Arabidopsis* that affect such marks lead to the release of TGS [1]. Thus transcriptionally silent or active states of chromatin are thought to be regulated by changes in DNA and by modifications of histones. Contradicting this general view, *mom1* mutations release silencing without obvious changes in DNA methylation, histone modification or degree of chromatin condensation [2],[3]. Analysis of genetic interactions between *mom1* and the *ddm1* mutation, which results in a severe decrease in DNA methylation and the relocation of histone modifications, suggested that MOM1and DDM1 act in independent but mutually reinforcing silencing pathways [4]. Moreover, DDM1 and MOM1 control TGS at overlapping targets that are reactivated when only a single pathway is compromised [5],[6]. Interestingly, a MOM1-specific subset of silencing targets has chromatin properties intermediate between hetero- and euchromatin. Thus, although silent these genes are poised for activation [6]. Similar bivalent chromatin properties have been found at several chromosomal loci in mammalian stem cells prior to their differentiation [7]. Unfortunately, mammalian epigenetic regulators responsible for controlling the transcriptional status of the intermediate chromatin have not been identified and MOM1 is the only example so far of a regulator determining the transcriptional status of targets associated with bivalent epigenetic marks.

MOM1 shares sequence homology with many proteins in a region containing a partial SNF2 domain [8]. SNF2 domains are found in ATP-dependent chromatin remodeling proteins involved in transcriptional control, DNA repair, and recombination. They contain seven conserved sequence motifs found in the superfamily II of DNA/RNA helicases [9]. The spatial structure of the SNF2 domain includes two lobes separated by a cleft [9]. The first lobe comprises helicase motifs I, Ia, II, III and the second includes motifs IV, V, and VI. Since the helicase motifs in the SNF2 sequence of MOM1 correspond only to the second lobe, Amedeo *et al.* (2000) [8] proposed that MOM1 functions as a heterodimer with an unknown *Arabidopsis* protein contributing the first SNF2 lobe.

The sequence close to the C-terminus of MOM1 shows similarity to an actin-binding domain (ABD) of chicken tensin [8]. Further predictions based on MOM1 protein sequence revealed a putative transmembrane domain, three putative nuclear localization signals (NLS) and several repetitive regions [8]. However, the functional relevance of all these sequence motifs was obscure.

In the present study, we demonstrate that a protein comprising 12.8% of the original MOM1 retains silencing activity through a novel motif necessary and sufficient for the MOM1 silencing function. The protein lacks all features previously considered important, except the NLS. MOM1-related proteins containing this new motif are present in the genomes of vascular plants but not in the mosses. Closer comparison of MOM1 orthologs suggests that MOM1 diverged, during the evolution of vascular plants, from the CHD3 chromatin remodeling factors common to many eukaryotes. We provide evidence that the two proteins are still able to cooperate in the control of TGS, despite the divergent evolution associated with the creation of a novel, MOM1-specific gene silencing domain and the degeneration of domains essential for CHD3 function.

## Results

### MOM1 Has Three Novel, Conserved Motifs

**Figure 1:**
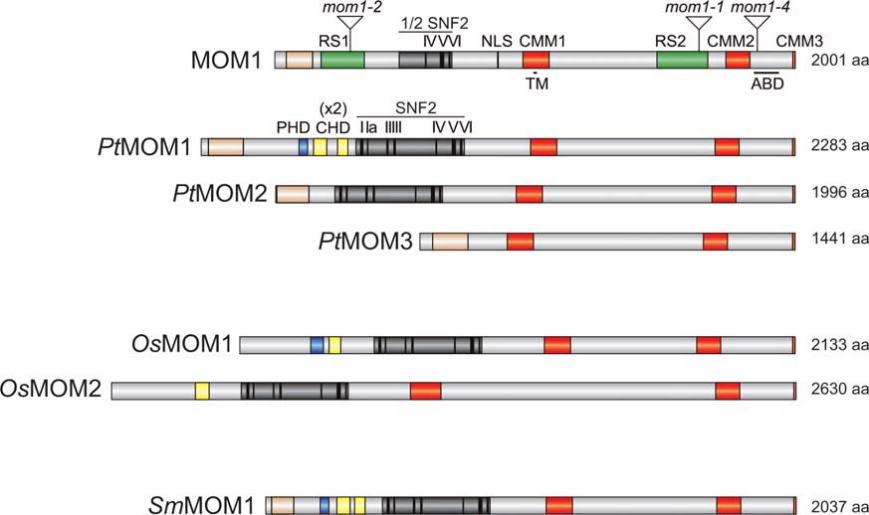
Schematic alignment of *Arabidopsis* MOM1 protein with its homologs in poplar (*Pt*MOM1-3), rice (*Os*MOM1-2) and club moss (*Sm*MOM1). Triangles indicate the positions of T-DNA insertions in the *Arabidopsis MOM1* gene: *mom1-1* (insertion after encoding 1633 aa, accompanied by a 2-kb deletion beyond the insertion site) [8], *mom1-2* (insertion after encoding 292 aa; SAIL_610_G01) and *mom1-4* (insertion after encoding 1860 aa; SALK_131757). MOM1 contains two repetitive sequences, RS1 and RS2 (green boxes), a nuclear localization signal (NLS), a putative transmembrane domain (TM), and a region similar to an actin-binding domain (ABD) of chicken tensin [8]. SNF2 domains are shown dark grey with black lines representing the conserved helicase motifs (I, Ia, II, III, IV, V and VI). The Conserved MOM1 Motifs (CMM1-3) are marked as red boxes. MOM1 shares homology with *Pt*MOM1-3 and *Sm*MOM1 in its N-terminal region (light yellow). Some MOM1 homologs additionally bear a plant homeodomain (PHD, blue) finger and one or two chromodomains (CHD, yellow). Predicted polypeptide size is shown on the right. Position of conserved domains: CMM1(953–1044 aa), CMM2(1734–1815 aa), CMM3 (1993–2001 aa) and N-terminal homology region (33–140 aa).

Earlier homology searches with MOM1 identified no other conserved sequences than the SNF2 domain and an actin-binding region [8]. Since then, many sequences have been added to databases and the genomes of rice (*Oryza sativa*) and poplar (*Populus trichocarpa*) have been sequenced and annotated [10],[11]. In the genomes of both these species, we have detected predicted proteins with MOM1 homologies extending beyond the SNF2 domain. In the poplar database, we detected three expressed proteins sharing homology with MOM1, referred to as *Pt*MOM1, *Pt*MOM2 and *Pt*MOM3 (Figure 1). In the rice database, we found two expressed MOM1 homologs, *Os*MOM1 and *Os*MOM2 (Figure 1). A gene encoding a predicted *MOM1* homologue was also found in the genome of the club moss (*Selaginella moellendorffii*), referred as *Sm*MOM1 (Figure 1). In addition to closely related SNF2 sequences, alignment of MOM1 and the homologs revealed three further conserved regions shared by these proteins that we named CMM1-3 (for Conserved MOM1 Motif 1-3) (Figure 1 and Figure S1). Noticeably, two poplar (*Pt*MOM1 and 2) and rice MOM1-related proteins encode complete SNF2 domains with all seven helicase motifs. Moreover, several MOM1 homologs contain additional sequence motifs such as a Plant Homeo Domain (PHD) and chromodomains (Figure 1).

As well as in Angiosperms, further database searches revealed CMM-containing proteins that could be predicted from not fully annotated genomic databases of more distant vascular plants like pine (*Pinus taeda*) (Figure S1 and data not shown). Remarkably, MOM1 homologs seem to be absent from *Chlamydomonas reinhardtii* and the moss *Physcomitrella patens*. The apparent conservation of additional MOM1-specific structural features might point towards a role in MOM1-mediated gene silencing. To address this issue, we assessed the functional significance of conserved MOM1 domains *in vivo*.

### Mutant Alleles of *MOM1* Define a Region Essential for MOM1 Function

**Figure 2:**
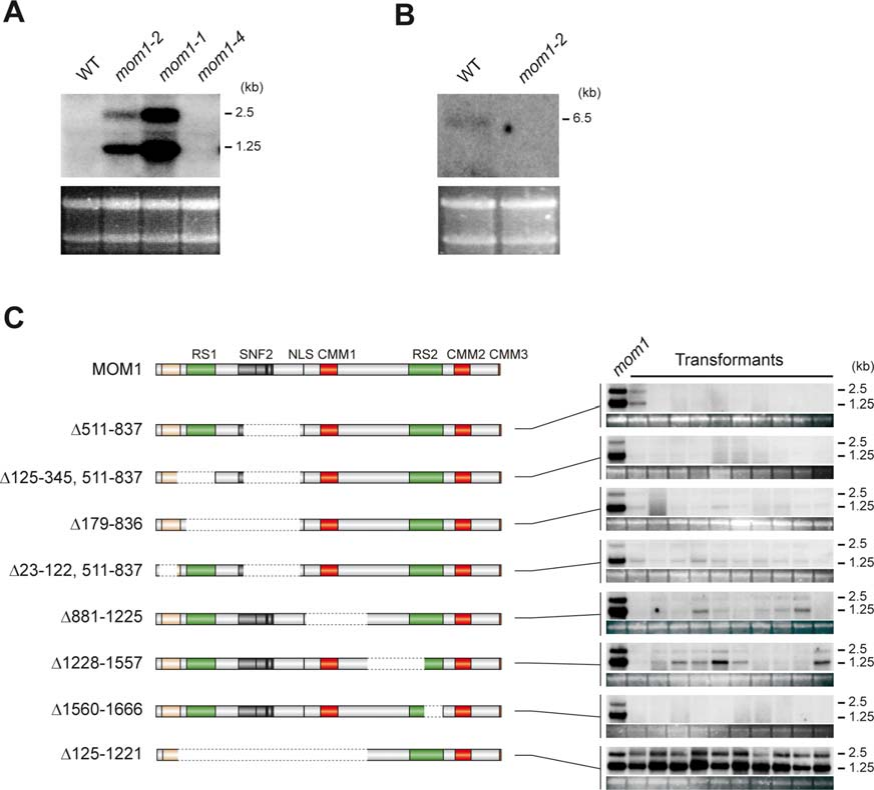
Functional analysis of *MOM1* deletions. (A) Levels of TSI transcripts in different mutant alleles of the *MOM1* gene (the different levels of TSI in *mom1-1* and *mom1-2* are due to different genetic background of mutants, Zurich and Columbia, respectively). (B) Northern blot showing the presence of the *MOM1* transcript in wild-type *Arabidopsis* and its depletion in the *mom1-2* mutant. Below the blots: ethidium bromide-stained RNA gel used for the blotting, as a loading control. (C) Top line: a schematic representation of the MOM1 protein marked as in Figure 1. Below: a schematic representation of series of deletion constructs (the deleted part is marked by dotted lines and specified on the left). Northern blot revealing levels of TSI transcripts in RNA samples isolated from *mom1* and 10 independent transgenic plants transformed with corresponding *MOM1* deletion derivatives are shown on the right (marked as “Transformants”). TSI transcripts in wild type *Arabidopsis* cannot be detected by Northern blots (see (A)). Ethidium bromide-stained RNA gels are shown below each Northern blot as a loading control. TSI transcripts sizes are indicated on the right.

Loss of silencing in the *mom1-1* mutant (Figure 1), which is predicted to encode a MOM1 protein with a deletion spanning the sequence 1633-2001aa (MOM1^Δ1633–2001^) [8], implies that the missing section is essential for MOM1 function. Plants homozygous for the *mom1-1* allele lose the ability to maintain TGS at previously silenced transgenic and endogenous chromosomal loci such as TSI (for Transcriptionally Silent Information) [3],[8]. In contrast, the previously uncharacterized *mom1-4* allele (Figure 1), which is predicted to encode the MOM1 C-terminal truncation of 142 amino acids (MOM1^Δ1860–2001^), is able to maintain TSI or transgenes silencing (Figure 2A and data not shown). To exclude the possibility that the T-DNA is spliced out of transcripts of the *mom1-4* locus, we performed both RT-PCR with primers corresponding to the *MOM1* sequence flanking the T-DNA insert and 3′RACE. The results from both assays were consistent with the termination of *MOM1* transcripts within the T-DNA insertion (data not shown). Thus, the sequence absent in *mom1-4*, notably including the ABD and CMM3 (Figure 1), is dispensable for MOM1 function in TGS. Contrasting phenotypes of *mom1-1* and *mom1-4* provide evidence that a functionally essential domain resides between amino acids 1633–1859. However, effects such as an altered protein structure or the reduced stability of the *mom1-1* gene product could not be ruled out. Moreover, it is possible that other parts of MOM1 are also essential for its function, given the presence of the 1633–1859 domain. To address these questions, we performed functional assessment of a series of *MOM1* gene deletions.

### SNF2 Helicase Motifs Are Dispensable for MOM1 Function

Although the partial SNF2 domain might be just a nonfunctional remnant of a complete domain of an MOM1 ancestor, as illustrated by the structures of MOM1-related proteins in poplar and rice, its presence may still be required for MOM1 silencing activity. To examine this, we tested a MOM1 deletion derivative lacking SNF2-related sequences as a substitute for the wild-type MOM1 protein. For this and the subsequent assays we used transgenic complementation tests of two *mom1* mutant alleles: *mom1-1* (MOM1^Δ1633–2001^) discussed above and *mom1-2* (Figure 1, Figure 2A). In *mom1-2*, the T-DNA insertion is predicted to result in truncation of more than 85% of the protein-coding sequence (T-DNA insertion after encoding 292 aa, Figure 1). Moreover, the MOM1 transcript is undetectable in *mom1-2* (Figure 2B). These features suggest that *mom1-2* is a null allele. When successful, the transgenic complementation tests should restore silencing of TSI sequences in the strains with *mom1* mutant alleles [3]. We introduced a modified *MOM1* gene encoding MOM1^Δ511–837^ lacking all three helicase motifs IV, V and VI into *mom1* mutants. This truncated *MOM1* gene re-established silencing of TSI (Figure 2C). The apparent dispensability of the SNF2 domain unequivocally demonstrates that this domain and thus any presumed chromatin remodeling activity is not involved in MOM1-mediated silencing.

### RS1, RS2, and CMM1 Are All Dispensable for the MOM1 Silencing Function

In order to assess the functional significance of other MOM1 protein sequence motifs, we performed systematic deletion/complementation analysis as for the SNF2 motif described above.

First, we constructed a series of deletions 5′ to the SNF2 region. This area encodes the first of the two repeated sequences (RS1) and an homologous region conserved between *Arabidopsis*, poplar and *Selaginella* but not present in rice MOM1 proteins (Figure 1). RS1 is composed of two repeats sharing higher similarity at the nucleotide (85.3%) than at the amino acid sequence level (78.0%) (data not shown), indicating a relatively recent duplication event. Both MOM1^Δ125–345,511–837^, lacking both RS1 and SNF2-related sequences, as well as MOM1^Δ179–836^, lacking RS1, the SNF2 domain and sequence linking those two elements, retained silencing activity (Figure 2C). Similarly, we assessed the functional importance of the N-terminal MOM1 sequence homologous to poplar MOM1- related proteins and *Selaginella* MOM1 (Figure 1). Since the predicted translation initiation of MOM1 is only five nucleotides after the first intron/exon junction, the most N-terminal deletion was introduced only after the first 22 amino acids of the predicted MOM1 sequence to ensure correct splicing. The resulting MOM1^Δ23–122,511–837^ was able to almost completely complement *mom1* mutants (Figure 2C). This demonstrates that the N-terminal conserved sequence is also dispensable for MOM1-mediated gene silencing or has very minor contribution.

We next constructed a series of deletions 3′ to the SNF2 region. This area encodes the second of the two repeated sequences (RS2) and three CMM motifs conserved in poplar, rice and other vascular plants (Figure 1, Figure S1, and data not shown). MOM1^Δ881–1225^ lacking CMM1 retained its silencing activity, indicating that CMM1 is also dispensable for TGS (Figure 2C). The silencing activity of MOM1^Δ881–1225^ contradicts the previously proposed hypothesis that MOM1 acts in association with the nuclear membrane, as the deletion encompasses also the previously predicted transmembrane domain [8]. The repetitive sequence RS2 was targeted in two constructs encoding MOM1^Δ1228–1557^ and MOM1^Δ1560–1666^ carrying successive deletions of two parts of RS2 (Figure 2C). Additionally, the MOM1^Δ1228–1557^ deletion covers a non-conserved sequence residing 3′ to CMM1 and 5′ to RS2. Successful complementation with all these constructs showed that this entire area of the gene is functionally dispensable.

### A Predicted NLS and CMM2 Are Essential for the MOM1 Silencing Function

Previous predictions based on the MOM1 sequence revealed three potential NLS sequences [8]; however, re-examination with recent NLS prediction algorithms [12] confirmed only one NLS (Figure 1). This NLS is bordered on both sides by functionally dispensable sequences examined in MOM1^Δ125–345,511–837^, MOM1^Δ179–836^ and MOM1^Δ881–1225^ proficient in TGS (Figure 2C). In contrast, MOM1^Δ125–1221^ with a large deletion encompassing the NLS together with surrounding, functionally superfluous sequences failed to complement *mom1-2* (Figure 2C). This suggests a requirement for NLS and is consistent with the reported nuclear localization of MOM1 [8].

**Figure 3:**
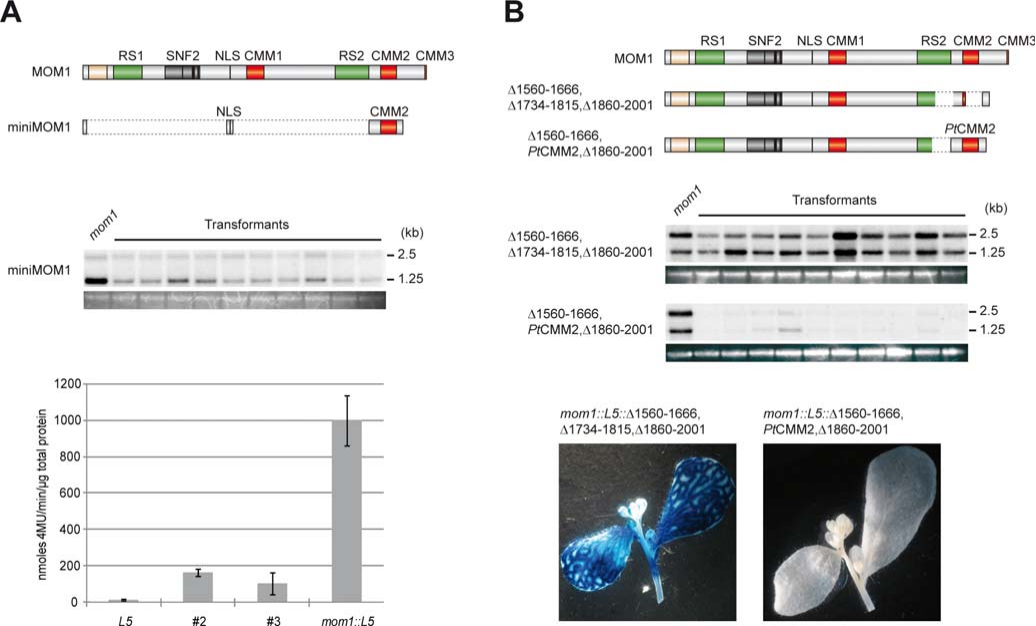
Evolutionary Conserved MOM1 Motif 2 (CMM2) is essential for MOM1 silencing function. (A) Top: schematic representation of “miniMOM1” compared with MOM1. Below: Northern blot displaying levels of TSI transcripts in RNA samples isolated from mom1 and 10 independent transgenic plants transformed with a “miniMOM1” construct. Bottom: Quantitative GUS expression for the resilencing of transgenic GUS locus of line L5, which being transcriptionally silent in wild-type Arabidopsis (*L5*) is activated in the *mom1* mutant (*mom1::L5*) and resilenced in the two independent transgenic lines transformed with a “mini MOM1” construct (*mom1::L5::miniMOM1* #2 and #3). The data are given as means of three independent assays, and error bars indicate standard error. (B) Top: schematic representation of MOM1^Δ1560–1666, 1734–1815, 1860–2001^ lacking CMM2 (Δ1560–1666, 1734–1815, 1860–2001) and MOM1^Δ1560–1666, 1734–1815, 1860–2001^ containing CMM2 from poplar (Δ1560–1666, *Pt*CMM2, Δ1860–2001). Below: Northern blots revealing levels of TSI transcripts in RNA samples isolated from *mom1* and 10 independent transgenic plants transformed with corresponding constructs. Bottom: histochemical staining for the resilencing of the transgenic GUS locus of line L5 by MOM1^Δ1560–1666, 1734–1815, 1860–2001^ with and without CMM2 from poplar. The histochemical assays shown are representative of plants from each genotype.

Successful complementation of *mom1* with a series of MOM1 truncations in the interval 22–1666 aa, with the exception of the 35 aa spanning the NLS, together with contrasting *mom1-1* and *mom1-4* silencing properties, suggest that the NLS and the sequence of 197 aa containing CMM2 are necessary and sufficient for the MOM1 silencing function. Therefore, we tested whether such a predicted “miniMOM1” protein (MOM1^Δ23–836,872–1662,1860–2001^), representing a fusion of these two sequences and comprising less than 13% of the MOM1, retains gene silencing activity (Figure 3A). The complementation tests clearly show that “miniMOM1” retains silencing activity, as reflected by a significant reduction in TSI transcription. However, TSI silencing seems to be incomplete and low levels of TSI RNAs were detected on Northern blots (Figure 3A). TSI consists of highly repeated elements residing in pericentromeric regions of all five *Arabidopsis* chromosomes. Therefore, it is possible that not all TSI templates are resilenced to completion by “miniMOM1” or that “miniMOM1” may require a minor contribution of conserved N-terminal sequence. Alternatively, the “miniMOM1” itself or its mRNA might be unstable and not able to reach levels allowing for complete TSI silencing. Therefore, we performed protein blots using material of randomly chosen transgenic lines. The “miniMOM1” protein was readily detected (Figure S2) and, thus, insufficient availability of “miniMOM1” cannot be considered as an explanation for incomplete TSI silencing.

To assess more precisely the silencing ability of “miniMOM1”, we introduced it into *mom1* mutant strains containing the silent GUS marker locus of line L5 [13]. In these strains, the mutations *mom1-1* or *mom1-2* release TGS of the GUS transgene (Figure 3A). The GUS transgene was, as TSI, almost completely resilenced upon introduction of “miniMOM1” (Figure 3A). These results confirm the silencing activity of “miniMOM1” and point to CMM2 as the main and possibly the only element clearly essential for the silencing activity of MOM1, given that the NLS is provided.

### MOM2 – a Non Functional *Arabidopsis* MOM1 Homolog

Available sequences indicate that the genomes of several plant species have genes encoding MOM1 homologs. In poplar, *Pt*MOM2 and *Pt*MOM3 represent truncated derivatives of PtMOM1 (Figure 1). Similarly, we found a transcribed gene in *Arabidopsis*, hereafter referred to as *MOM2* (At2g28240), predicted to encode a protein homologous to the C-terminal part of MOM1. MOM2 retained CMM2 and CMM3; however, it acquired a novel tandemly repeated sequence (RS). The absence of corresponding repeats in MOM1, along with presence of RS2 missing in MOM2, implies that these repeats were acquired independently after *MOM1* and *MOM2* diverged from a common ancestor. MOM2 lacks NLS and, furthermore, its CMM2 bears mutations in amino acids conserved in other MOM1 homologs (Figure S3 and data not shown). The two tested *mom2* mutant alleles *mom2-1* (WiscDsLox364H07) and *mom2-2* (SAIL548_H02) did not affect TSI silencing (Figure S3). Additionally, *mom1 mom2* double mutants had a level of TSI expression similar to that in *mom1* (Figure S3), indicating that MOM2 has no silencing function redundant with MOM1. These observations are in agreement with the essential roles of the NLS and the intact CMM2 for gene silencing of MOM1.

### CMM2 Silencing Function Is Evolutionary Conserved

CMM2 was detected as one of three regions of MOM1-related proteins that are conserved in addition to SNF2 motifs (Figure 1 and Figure S1). To examine whether this structural conservation also reflects conservation of a silencing function, we replaced CMM2 of *Arabidopsis* MOM1 by the CMM2 predicted for *Pt*MOM1 of poplar (Figure 3B). We compared *mom1-2* complementation ability of the MOM1^Δ1560–1666, 1734–1815, 1860–2001^ construct lacking CMM2 to the same construct containing CMM2 from poplar. Poplar CMM2 clearly restored the silencing activity of MOM1^Δ1560–1666, 1734–1815, 1860–2001^, suggesting that *Pt*MOM1 is able to perform gene silencing mediated by its CMM2 (Figure 3B).

### MOM1-Related Proteins Originate through Neo-Functionalization of Evolutionary Conserved CHD3-Like Silencing Factors

**Figure 4:**
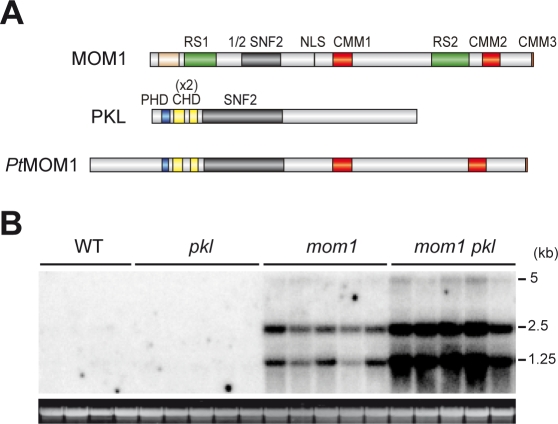
MOM1 and PKL together contribute to the control of TGS. (A) Schematic comparison of predicted protein domains of MOM1, PKL and *Pt*MOM1 (color code as in Figure 1). (B) Northern blot showing levels of TSI transcripts of four wild-type plants (WT) and five plants of each mutant strain; *pkl*, *mom1* and *mom1pkl* double mutant. Below the blot: ethidium bromide-stained RNA gel used for the blotting as a loading control.

The sequence of *Pt*MOM1 also predicts, in addition to an integral SNF2 domain with all six helicase motifs, the presence of a PHD finger and double chromodomains (Figure 1). The combination of PHD fingers, double chromodomains and an SNF2 domain is a distinctive feature of CHD3 proteins (Chromodomain-Helicase-DNA binding) [14]; noticeably plant CHD3-like proteins retained only a single PHD finger domain. The intact SNF2 domain is critical for the silencing function of CHD3 proteins. The long life-span of poplar and continuous production of “ancient” gametes is thought to reduce significantly the speed of genome evolution compared with *Arabidopsis* (estimated at six times) [11]. Therefore, *Pt*MOM1 presumably reflects a more ancient sequence arrangement than those of the *Arabidopsis* or rice MOM1 proteins and the presence of all CHD3 domains in *Pt*MOM1 provides strong support for an evolutionary link between MOM1 and CHD3 proteins (Figures 1, 4 and Figure S4). *Pt*MOM1structural features were also found in *Sm*MOM1 (Figure 1) providing additional support to this conclusion.

The *Arabidopsis* genome contains two genes encoding CHD3-like proteins – *PICKLE* (*PKL*) (Figure 4A) and the as yet uncharacterized At5g44800. PKL is required for postembryonic transcriptional suppression of genes involved in embryogenesis [15],[16] and seems to contribute also to the restriction of ectopic meristematic activity [17].

### *Arabidopsis* MOM1 and PKL Together Contribute to TGS Control

Since *MOM1* and *PKL* likely diverged from a common ancestral *CHD3*-like gene, we were interested to examine whether their functions may still converge in the control of gene silencing. We combined the *pkl* and *mom1* mutations and compared levels of transcriptional reactivation of TSI in the single and *pkl mom1* double mutants (Figure 4B). TSI activated in *mom1* remained silent in *pkl*, suggesting that, in contrast to MOM1, depletion of PKL was not sufficient to release TSI silencing. However, the level of TSI transcripts was increased approximately fourfold in *pkl mom1* double mutants compared with the *mom1* single mutant. Thus, even though PKL and MOM1 diverged in terms of their active domains, they are still able to cooperate functionally in the control of TGS.

## Discussion

Unexpectedly, we have found that more than 87% of MOM1 protein is dispensable for the gene silencing function, according to the functional analysis of a series of deletion derivatives of the *MOM1* gene. We have also demonstrated that a “miniMOM1”, comprising 22 N-terminal amino acids, an NLS and 197 amino acids including CMM2, retains silencing activity, as reflected by drastically reduced levels of TSI expression and almost complete transcriptional suppression at a transgenic GUS locus. Therefore, minor contribution of the N-terminal part of MOM1 to its silencing activity seems to be apparent. In addition, a drastic reduction in protein size leading to alterations in physical properties (e.g. a predicted isoelectric point of 5.2 for MOM1 and 8.8 for “miniMOM1” and a change in net charge from −62 to +4.3) can also contribute to the incomplete silencing mediated by “miniMOM”. Nevertheless, the results of MOM1 deletion analysis and the successful replacement of *Arabidopsis* CMM2 by the CMM2 of poplar provide strong evidence that CMM2 is the most critical element of the MOM1 protein for its silencing function, not only in *Arabidopsis* but also in other plants. Obviously other domains, also these clearly dispensable for TSI and transgene silencing, may still be required for epigenetic regulation at other, as yet unidentified, target loci.

Although, MOM1 proteins are CHD3 derivatives, the domains shared with CHD3 chromatin remodeling factors apparently became obsolete after the acquisition of CMM2. This is also evident for *Pt*MOM1, which has a structure largely similar to CHD3 proteins. For example, the SNF2 domain of *Pt*MOM1, shown to be critical for the function of CHD3 proteins, acquired mutations of conserved amino acids essential for CHD3 activity [15],[18],[19] (Figure S5). Several indispensable amino acids are replaced in MOM1 homologs from different plant species and, remarkably, these replacements are identical in MOM1 proteins from different plant species. It is difficult to provide a simple explanation for this unusual sequence drift since the loss of remodeling functions of SNF2 should not be under a direct, strong selection pressure for particular types of mutations. In any case, the pattern of these mutations provides specific signatures to MOM1 SNF2 domains (Figure S5 and S6) and suggests that acquisition of CMM2 and degeneration of SNF2 occurred in species ancestral to vascular plants. The SNF2 domain of *Arabidopsis* MOM1 underwent the most drastic alterations due to an internal deletion. This relatively recent event seems to be accompanied by the formation of the RS1 sequence duplication. Alignment of *Arabidopsis* and poplar sequences flanking RS1 suggests that extensive deletion and the formation of RS1 removed not only part of the SNF2 domain but also a PHD finger and chromodomains. Clearly, this event provides the best illustration of the dispensability also of the PHD finger, and chromodomain for the MOM1 silencing function.

CHD3 proteins of human and *Drosophila*, known as Mi-2, act as components of a multi-subunit chromatin repression complex NuRD (Nucleosome Remodelling and Deacetylating), which combines nucleosome remodelling and histone deacetylation activities [20],[21]. The *Arabidopsis* genome encodes two CHD3-like proteins: PKL with a potentially functional SNF2 domain and the still uncharacterized At5g44800 with an SNF2 domain containing mutations in amino acids essential for chromatin remodeling activity (Figures S5, S6 and data not shown). PKL is involved in transcriptional repression of genes that are active only at a particular time and place during sporophyte development [15],[16],[17],[22]. However, there is little evidence at present for the involvement of chromatin remodeling and histone deacetylation in PKL-mediated gene repression.

The exact mechanism of MOM1-mediated silencing is not known, but MOM1 and PKL both seem to contribute to transcriptional suppression or restriction of levels of ectopic reactivation of TSI transcription. The multilayer nature of epigenetic regulation and the necessity for backup mechanisms have been documented recently for *Arabidopsis* gene silencing associated with DNA methylation changes [23]. However, in this case, interaction between the major and evolutionary highly conserved gene silencing mechanisms, such as DNA and histone methylation, was investigated and the backup deficiencies were found to have very drastic developmental consequences indicative of the destabilization of central epigenetic functions. The effects of *pkl* or *mom1* and the combination of these mutations have much more subtle effects. This can be explained by the characteristics of MOM1 targets and their association with bivalent epigenetic marks. The number of such loci is low [6] and their reactivation is likely controlled at multiple levels, as illustrated here by the cooperative activities of MOM1 and PKL. It is remarkable that despite the clearly divergent evolution of MOM1 in terms of protein properties, it has retained its functional relationship to the CHD3 proteins. The CHD3 origin of MOM1 and the silencing in cooperation with PKL suggest that MOM1 function is also linked to histone acetylation changes. Although global changes in histone acetylation properties were not observed in *mom1* mutants [2], more subtle target-specific acetylation changes cannot be ruled out.

Whatever the precise molecular mechanism(s) of heritable transcriptional repression mediated by MOM1 might be, it is remarkable that increasing complexity of epigenetic gene regulation has resulted from the emergence of supplementary and cooperating levels and/or mechanisms of epigenetic control. Genomic or cDNA sequences encoding CMM2 are present in many species of vascular plants, even as distant as club-moss *Selaginella moellendorffii*, pine *Pinus taeda* and various monocotyledonous and dicotyledonous species. Remarkably, we failed to detect CMM2 in the moss *Physcomitrella patens* or in green algae *Chlamydomonas reinhardtii*, although the sequences of both genomes are complete (http://genome.jgi-psf.org/). Therefore, it appears that the emergence of CMM2, and thus MOM1 proteins, coincided with the appearance of vascular plants. Since MOM1-related proteins are not present outside of the plant kingdom, it can be envisaged that novel, highly specialized epigenetic factors and functions can appear only in a narrow subset of organisms through diversification of the general, evolutionary conserved epigenetic regulators. So far the biological role of the CHD3/MOM1 sub-diversification remains unclear but it is intriguing that it seems to have assisted the major evolutionary step in the emergence of land plants.

## Materials and Methods

### RNA Analysis

Total RNA was isolated using the TRI reagent (Sigma) according to the manufacturer's instructions. Detection of TSI was as described previously [3].

### Gene Manipulation and Transformation of Plants

The MOM1 genomic sequence was assembled in the binary vector pCAMBIA1301 after elimination of vector sequence EcoRI-BstEII encompassing a multiple cloning site and the β-glucuronidase gene. DNA fragments covering the entire MOM1 gene and ∼2 kb of sequence upstream of the transcription start were detected in the *Arabidopsis thaliana* Lambda Genomic Library (Stratagene) and assembled in the modified vector.

To create MOM1^Δ511–837^, MOM1^Δ1096–1234^, MOM1^Δ881–1225^, MOM1^Δ125–345,511–837^, MOM1^Δ179–836^, MOM1^Δ125–1221^, MOM1^Δ1228–1557^, MOM1^Δ1560–1666^, MOM1^Δ23–122,511–837^, MOM1^Δ23–836,872–1662,1860–2001^, the MOM1 gene sequences bordered by restriction sites *Bst*BI-*Xma*I, *Ase*I-*Ase*I, *Xma*I-*Bbv*CI, *Sal*I-*Bst*BI, *Sal*I-*Xma*I, *Sal*I-*Bbv*CI, *Bbv*CI-*Blp*I, *Blp*I-*Blp*I, *Nco*I-*Sal*I, *Nco*I-*Bst*BI, respectively, were replaced with oligonucleotide adapters or amplified fragments containing matching restriction sites. To create MOM1^Δ125–345,511–837^, MOM1^Δ179–836^, MOM1^Δ23–122,511–837^ and MOM1^Δ23–836,872–1662,1860–2001^, the corresponding deletions were introduced into MOM1^Δ511–837^. Constructs encoding the modified MOM1 proteins were introduced into *mom1* mutant plants using the floral dip method [24].

### Western Blotting

A total protein extract in Laemmli sample buffer was fractionated by 10% SDS/PAGE and blotted onto a Hybond-P membrane (Amersham Pharmacia). Proteins were visualized using the ECL PLUS kit (Amersham Pharmacia) after membrane hybridization with anti-HA antibody (Roche).

### GUS Assays

Staining was performed on 1-week-old seedlings as described [25]. Quantitative GUS activity assay was performed on 13-day-old plantlets as described [25] with minor modifications.

### Accession Numbers

*AtMOM1* (At1g08060); *AtMOM2* (At2g28240); *PtMOM1* (eugene3.00130053); *PtMOM2* (eugene3.00660276); *PtMOM3* (eugene3.01310088); *OsMOM1* (Os06g01320); *OsMOM2* (Os02g02050); *SmMOM1* (estExt_fgenesh2_pg.C_110182); *PKL* (At2g25170).

## Supporting Information

Figure S1 Multiple alignments of CMM2s encoded in predicted proteins from various plant species. At - *Arabidopsis thaliana*, Pt - *Populus trichocarpa*, Vv - *Vitis vinifera*, Os - *Oryza sativa*, Pta - *Pinus taeda*, Zm - *Zea mays*, Af - *Aquilegia formosa*, Cs - *Citrus sinensis*, Mt - *Medicago truncatula*, Sm - *Selaginella moellendorffii*. “*” identical residues; “:” conserved substitutions.
(0.02 MB PDF)

Figure S2 Detection of HA-tagged miniMOM1 protein by Western blots in extracts of transgenic T1 plants transformed with the miniMOM1 construct depicted in Figure 3. Below: Coomassie blue-stained gel with identical samples, as a loading control.
(0.05 MB PDF)

Figure S3 MOM2. Top: schematic representation of predicted MOM2 protein of *Arabidopsis* (triangles mark insertion sites of T-DNA in *mom2-1* and *mom2-2* mutants). CMM2 bears mutations in amino acids conserved in other MOM1 homologs (represented by white stripes). Below: Northern blot revealing the levels of TSI transcripts in *mom1* and *mom2* mutants and double mutants. Below: the blot ethidium bromide-stained RNA gel used for the blotting, as a loading control.
(0.16 MB PDF)

Figure S4 Maximum likelihood tree of chromodomain 1 and 2 (CHD1, CHD2) amino acid sequences. The sequences were aligned using the Seaview program [26]. The variable regions were removed and 45 sites were retained for analyses. The maximum likelihood tree was inferred using Treefinder program [27] with WAG+G (4 categories) model. The similar topology was obtained using neighbor joining method, as implemented in PhyloWin program [26], with as the only differences the position of PKL_CHD2 branching out of the clade HsCHD3CHD2+DmMi2CHD2 and the changes in the branching order within MOMCHD1 clade. The numbers at internal nodes indicate bootstrap values for ML and NJ analyses.
(0.02 MB PDF)

Figure S5 Alignment of SNF2 domains from MOM1 homologues and other SNF2-containing proteins. The conserved helicase motifs are framed in red. Point mutations of conserved amino acids that are known to inactivate SNF2 domains of dMi-2 [18],[28], PKL [17] and SYD [29] are indicated above the alignment. Asterisks below the alignment indicate amino acids conserved in MOM1 homologues but absent from other SNF2-containing proteins.
(0.04 MB PDF)

Figure S6 Maximum likelihood tree of SNF2 amino acid sequences. The sequences were aligned as indicated at text-Figure using the Seaview program [26]. The variable regions were removed and 211 out of 240 amino acid sites were retained for analyses. The maximum likelihood tree was inferred using Treefinder program [27] with WAG+G (4 categories) model. The similar topology was obtained using neighbour joining method, as implemented in PhyloWin program [26], except that AtMOM branches with PtMOM in the NJ tree. The numbers at internal nodes indicate bootstrap values for ML and NJ analyses.
(0.02 MB PDF)
