# Supplementary figures and images for: Divergent Evolution of CHD3 Proteins Resulted in MOM1 Refining Epigenetic Control in Vascular Plants
Source: PLoS Genet. 2008 Aug 22;4(8):e1000165. doi: 10.1371/journal.pgen.1000165 (PMC2507757; doi:10.1371/journal.pgen.1000165)

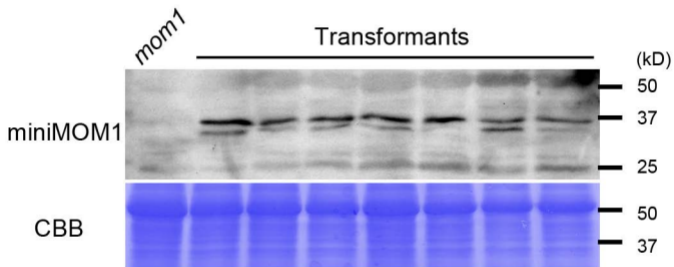

Supplement: Figure S2 — Detection of HA-tagged miniMOM1 protein by Western blots in extracts of transgenic T1 plants transformed with the miniMOM1 construct depicted in Figure 3. Below: Coomassie blue-stained gel with identical samples, as a loading control. (0.05 MB PDF) [file pgen.1000165.s002.pdf]

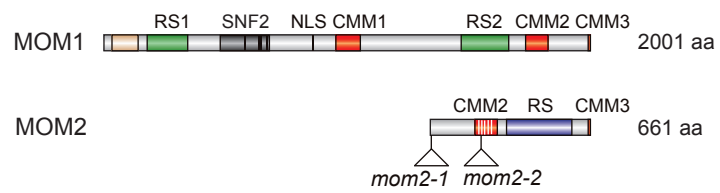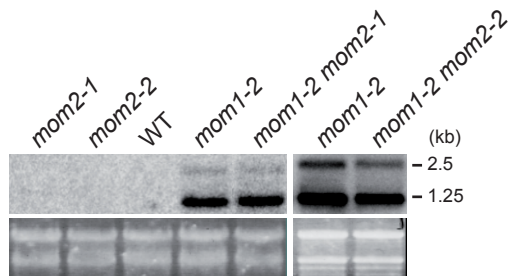

Supplement: Figure S3 — MOM2. Top: schematic representation of predicted MOM2 protein of Arabidopsis (triangles mark insertion sites of T-DNA in mom2-1 and mom2-2 mutants). CMM2 bears mutations in amino acids conserved in other MOM1 homologs (represented by white stripes). Below: Northern blot revealing the levels of TSI transcripts in mom1 and mom2 mutants and double mutants. Below: the blot ethidium bromide-stained RNA gel used for the blotting, as a loading control. (0.16 MB PDF) [file pgen.1000165.s003.pdf]

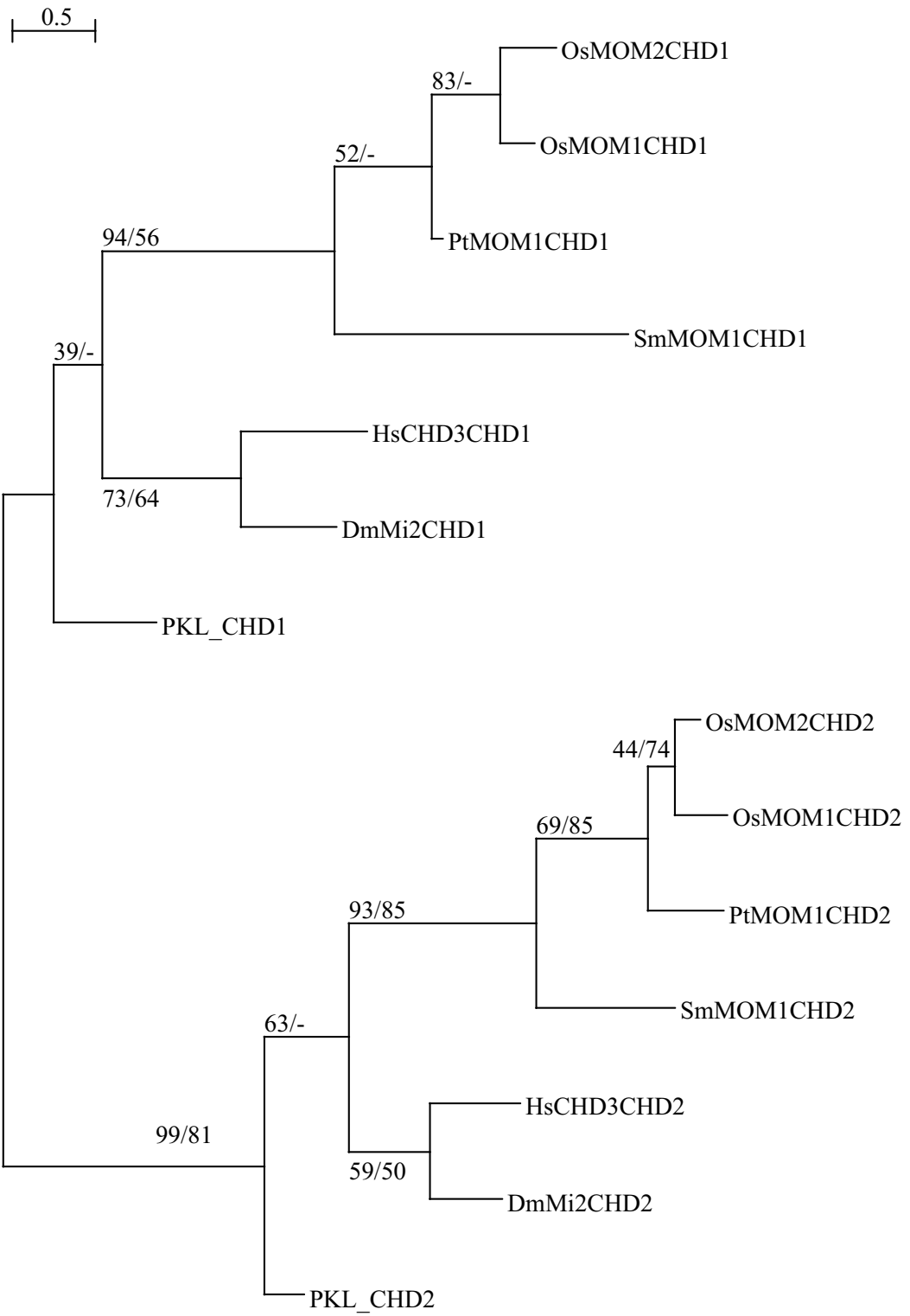

Supplement: Figure S4 — Maximum likelihood tree of chromodomain 1 and 2 (CHD1, CHD2) amino acid sequences. The sequences were aligned using the Seaview program [26]. The variable regions were removed and 45 sites were retained for analyses. The maximum likelihood tree was inferred using Treefinder program [27] with WAG+G (4 categories) model. The similar topology was obtained using neighbor joining method, as implemented in PhyloWin program [26], with as the only differences the position of PKL_CHD2 branching out of the clade HsCHD3CHD2+DmMi2CHD2 and the changes in the branching order within MOMCHD1 clade. The numbers at internal nodes indicate bootstrap values for ML and NJ analyses. (0.02 MB PDF) [file pgen.1000165.s004.pdf]

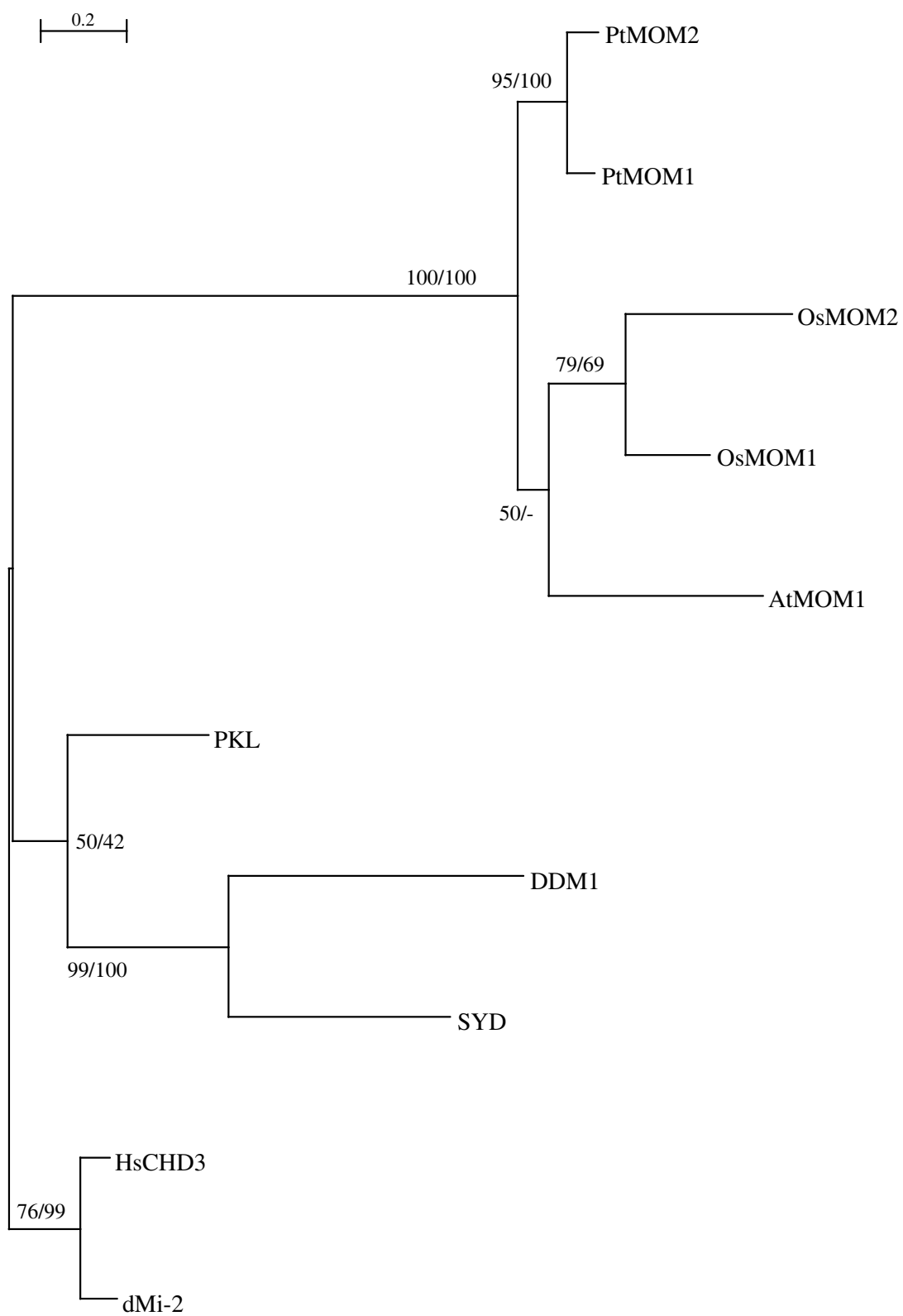

Supplement: Figure S6 — Maximum likelihood tree of SNF2 amino acid sequences. The sequences were aligned as indicated at text-Figure using the Seaview program [26]. The variable regions were removed and 211 out of 240 amino acid sites were retained for analyses. The maximum likelihood tree was inferred using Treefinder program [27] with WAG+G (4 categories) model. The similar topology was obtained using neighbour joining method, as implemented in PhyloWin program [26], except that AtMOM branches with PtMOM in the NJ tree. The numbers at internal nodes indicate bootstrap values for ML and NJ analyses. (0.02 MB PDF) [file pgen.1000165.s006.pdf]
