# Supplementary material for: Divergent Evolution of CHD3 Proteins Resulted in MOM1 Refining Epigenetic Control in Vascular Plants
Source: PLoS Genet. 2008 Aug 22;4(8):e1000165. doi: 10.1371/journal.pgen.1000165 (PMC2507757; doi:10.1371/journal.pgen.1000165)
Supplement: Figure S5 — Alignment of SNF2 domains from MOM1 homologues and other SNF2-containing proteins. The conserved helicase motifs are framed in red. Point mutations of conserved amino acids that are known to inactivate SNF2 domains of dMi-2 [18],[28], PKL [17] and SYD [29] are indicated above the alignment. Asterisks below the alignment indicate amino acids conserved in MOM1 homologues but absent from other SNF2-containing proteins. (0.04 MB PDF) [file pgen.1000165.s005.pdf]

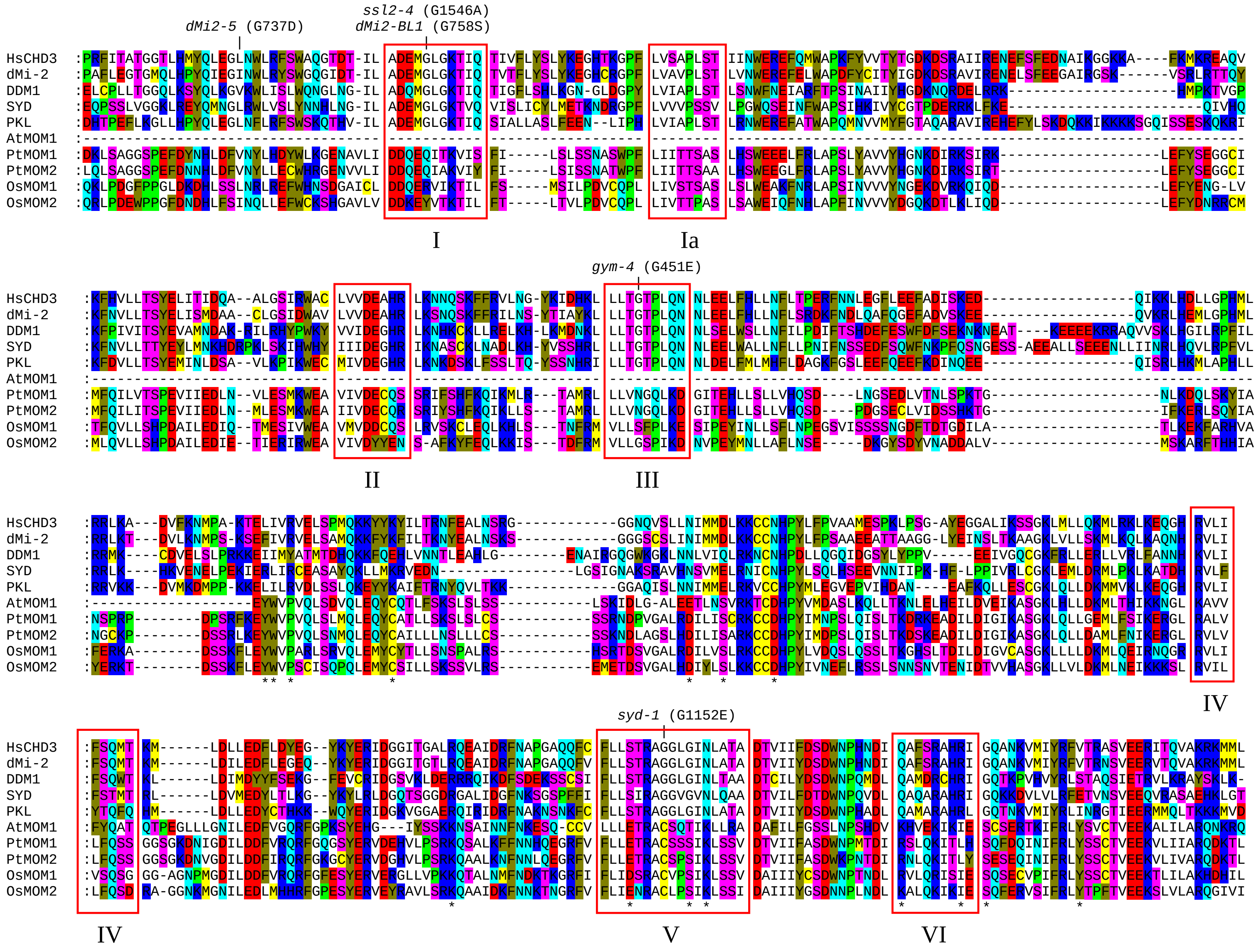
ssl2-4 (G1546A)
dMi2-5 (G737D)
dMi2-BL1 (G758S)
HsCHD3
dMi-2
DDM1
SYD
PKL
AtMOM1
PtMOM1
PtMOM2
OsMOM1
OsMOM2
I
Ia
gym-4 (G451E)
II
III
IV
syd-1 (G1152E)
IV
V
VI
